# Efficient Extraction of a Docosahexaenoic Acid (DHA)-Rich Lipid Fraction from *Thraustochytrium* sp. Using Ionic Liquids

**DOI:** 10.3390/ma11101986

**Published:** 2018-10-15

**Authors:** Yujie Zhang, Valerie Ward, Dorothy Dennis, Natalia V. Plechkova, Roberto Armenta, Lars Rehmann

**Affiliations:** 1 Department of Chemical and Biochemical Engineering, The University of Western Ontario, London, ON N6A 3K7, Canada; yzha2336@uwo.ca; 2 Department of Chemical Engineering, The University of Waterloo, Waterloo, ON N2L 3G1, Canada; vward@uwaterloo.ca; 3 Mara Renewables, Dartmouth, NS B2Y 4T6, Canada; ddennis@maracorp.ca (D.D.); rarmenta@maracorp.ca (R.A.); 4 QUILL, School of Chemistry and Chemical Engineering, Queen’s University Belfast, Belfast BT7 1NN, UK; n.plechkova@qub.ac.uk; 5 Department of Process Engineering & Applied Science, Dalhousie University, Halifax, NS B3H 4R2, Canada

**Keywords:** omega 3, ionic liquids, microalgae

## Abstract

Polyunsaturated fatty acids (PUFAs) play a significant role in the modulation and prevention of various diseases, and hence are attracting increasing attention from the biotech industry. Thraustochytrids are marine heterokonts that exhibit robust growth rates, high PUFA content, and more specifically, a large percentage of omega-3 fatty acids like docosahexaenoic acid (DHA). Recently, ionic liquids (ILs) have been shown to improve the efficiency of organic solvent extraction of oils from wet oleaginous yeast and microalgae under mild conditions. Two ILs, the imidazolium 1-ethyl-3-methylimidazolium ethylsulfate [C_2_mim][EtSO_4_] IL and the phosphonium (tetrabutylphosphonium propanoate [P_4444_][Prop]) IL were assessed for their ability to facilitate extraction of PUFA-containing lipids from a *Thraustochytrium* sp. (T18) through efficient cell wall disruption. The oil extracted after IL pretreatment was further characterized with respect to fatty acid methyl ester (FAME) composition, while the effects of process parameters, such as the ratio of ionic liquid to co-solvent, the mass ratio of microalgae to the mixture of ionic liquid, and type of co-solvent were also investigated for both ILs. The results indicate that these ILs can disrupt the cells of *Thraustochytrium* sp. when mixed with a co-solvent (methanol), and facilitated the recovery of oils over a large degree of dewatered *Thraustochytrium* biomass (0–77.2 wt% water) in a short period of time (60 min) at ambient temperature, hence demonstrating a water compatible, low-energy, lipid recovery method. The lipid recovery was not affected by repeated usage of recycled ILs (tested up to five times).

## 1. Introduction

The consumption of omega-3 polyunsaturated fatty acids (PUFAs), such as docosahexaenoic acid (DHA, 22:6n-3) or eicosapentaenoic acid (EPA, 20:5n-3), has been linked to the prevention of neural disorders, cardiovascular diseases, arthritis, asthma, and dermatosis [1,2,3,4]. PUFAs are involved in many vital physiological functions and are important components of brain cell membranes [5,6,7]. Their possible use in reducing cholesterol when consumed as food supplements has made their production for nutritional purposes highly desirable. For the extraction of PUFAs, data from various types of biomass can be found in the literature. Routray et al. obtained 19.18 g/100 g oil from salmon by-products using enzymes during the extraction process, and omega-3 PUFAs constituted a significant part [8]. Kuo et al used homogenization plus sonication to extract EPA/DHA-enriched oil from cobia liver, with 5.2% EPA and 19.7% DHA [9]. Oliveira et al. adopted enzymatic hydrolysis to extract oil from yellowfin tuna, which presented high levels of EPA (6.05 g/100 g) and DHA (27.15 g/100 g) [10]. Currently DHA is mainly produced from marine fish oil, but this route is becoming increasingly challenging due to marine pollution and seasonal variations in fish production [3,11]. Therefore, alternative sources of oils rich in PUFAs are being developed that aim to reduce the processing costs, increase the sustainability of their production, and reduce their environmental burden. One promising source of PUFAs are the heterokonts called thraustochytrids that exhibit robust growth rates and can accumulate high oil contents, particular of DHA, which can account for over 30% of the total cellular lipids [12,13,14]. Commonly used species belong to the *Aurantiochytrium*, *Schizochytrium*, *Thraustochytrium*, and *Ulkenia* genera [14]. In addition to their high PUFA content, thraustochytrids are capable of breaking down complex organic materials and using them as their main carbon source [15,16,17], making them easy to be cultivated on a wide range of feedstocks, including waste materials such as wastewater [14,18].

Like other sources of single-celled oils (SCOs), thraustochytrids are cultivated in aqueous media and current organic solvent extraction processes require energy-intensive and time-consuming drying steps to prepare the biomass for extraction [14]. Harvesting, drying, and extraction steps can account for up to 70% of the processing costs for SCOs [19,20]. Enhancing extraction efficiency depends on increasing the interfacial area of cellular matrix to solvent through cell disruption [20]. Cell disruption can be accomplished through a variety of mechanisms, many of which are time-consuming or energy-intensive. These include mechanical methods (comminution, ultrasonication, and high-pressure homogenization) [21,22], physical methods (thermochemical wall-breaking technology, microwaves, and repetitive freeze-thaw cycles) [23,24] and biochemical methods (the acid-heated method, the alkali-heated method, and enzyme hydrolysis) [25,26,27]. The diversity of the composition of the cell walls of heterokonts make it difficult to predict the application of one process to a new species. *Thraustochytrids* possess a laminated cell wall made of scales that are predominately protein-based (30–43%) with some carbohydrate content (21–36%) [28], while other fatty acid producing algae, such as diatoms, could have a silica cell wall [29]. As a result, assessing developing technologies on many species of oleaginous biomass is necessary to determine their applicability. Therefore, the commercial viability of *thraustochytrid*-based lipid production will be greatly improved by assessing new cell disruption techniques and developing processes that improve water compatibility and reduce the overall energy requirements of these costly down-stream processing steps.

Ionic liquids (ILs) have been shown to increase the lipid extraction efficiency from microalgae by dissolving both dry and wet microalgae under relatively mild conditions (80–140 °C) with and without a co-solvent (Table 1) [1,30,31]. ILs are often described as “green” designer solvents with many desirable physical-chemical properties such as great thermal stability, low melting points, and negligible vapor pressure [32]. In bioprocessing, ILs are best known for the capability of some ionic liquids to solvate highly recalcitrant biopolymers like cellulose [33,34,35].

**Figure 1:**
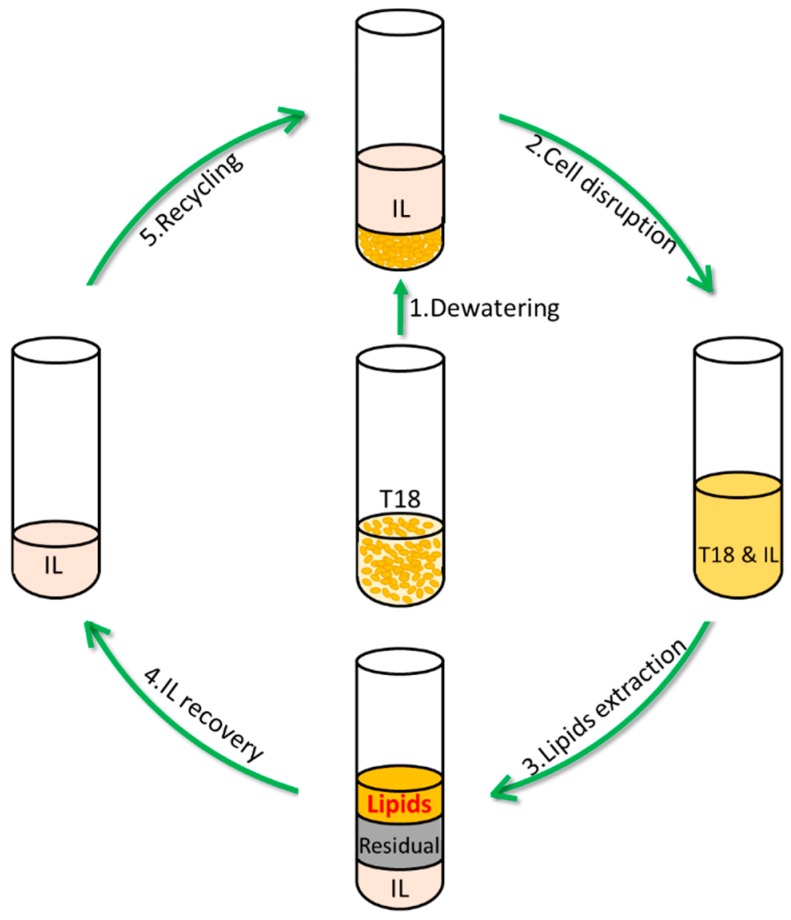
Flowchart of the lipid extraction and IL recycling.

Until now, imidazolium ILs have been the main focus for the cellular disruption of microalgae and other SCOs, likely owing to their greater commercial availability [20,36,37]. In most cases, hexane/2-propanol (H2P; 3:2 *v*/*v*) extraction or chloroform/methanol (2:1 *v*/*v*) was used for the analytical determination of the theoretical maximum lipid yield [20,30,38]. In this work two ILs were assessed for their ability to improve lipid-extraction from the *thraustochytrid* sp., T18 by disrupting the cell wall. Both dry and wet T18 was pretreated with either 1-ethyl-3-methylimidazolium ethylsulfate [C_2_mim][EtSO_4_] or tetrabutylphosphonium propionate [P_4444_][Prop] for cell wall disruption and lipids were briefly extracted using hexane. Extracted oils were further characterized for their lipid composition. Figure 1 presents the methodology employed for oil extraction from dewatered T18 biomass using ILs and the subsequent IL recycling process.

## 2. Materials and Methods

### 2.1. Materials and Strain

[C_2_mim][EtSO_4_] was purchased from Sigma-Aldrich (Oakville, ON, Canada). [P_4444_][Cl] donated by Solvay (Niagara Falls, ON, Canada) was used to synthesize [P_4444_][prop] by anion exchange with a sodium salt of propanoic acid and the structure was confirmed using standard methods [39]. *Thraustochytrium* sp. (T18) was obtained from the Canadian Phycological Culture Center (CPCC) (Waterloo, ON, Canada) strain PTA-6245. The cultivation conditions are described elsewhere [14]. 

### 2.2. Harvesting and Freeze-Drying

T18 cultures were harvested by centrifugation at 3500 rpm at 4 °C in a Sorvall RT centrifuge (Fisher Scientific, Ottawa, ON, Canada) for 20 min. Residual salts were removed from the cell pellets by washing three times with deionized water by repeated resuspension and centrifugation. After that, the cell pellets were frozen at −80 °C for a minimum of 12 h and then lyophilized using a 4.5 L freeze-drier (Labconco, Kansas City, MO, USA) for 24 h and stored in a desiccator until further use. For wet extractions, fresh T18 was harvested by centrifugation and resuspended in deionized water as above. After the last centrifugation, the T18 slurry was kept for the next extraction step. Dry weight was determined by overnight drying in an oven at 60 °C.

### 2.3. Analytical Determination of Total Lipid Content

The standard total lipid content was determined in triplicate by adding 0.1 g of freeze-dried algae to 5 mL of hexane/2-propanol solution (H2P; 3:2 *v*/*v*) [40] and stirring for 12 h. Then, the mixture was filtered through a Buchner funnel with a fine porosity fritted disc. The residual solids were washed with acetone until they were colorless. The appropriate phase was then transferred to a pre-weighed foil pan in a fume hood to evaporate the solvent. The mass of extracted lipids was measured using an analytical balance until the weight no longer fluctuated. When extracting from wet biomass, the first step was to concentrate the biomass via centrifugation. The wet slurry was washed three times with DI water to eliminate the residual media and salt. The resulting slurry was the raw material for extraction via the same operation steps as mentioned above.

### 2.4. IL Pretreatment 

IL pretreatments were done in triplicate by mixing 0.1 g of freeze-dried algae with the indicated mass of IL in tubes for 1 h with a magnetic stirrer at ambient temperature. Then 5 mL of hexane was added to the IL T18 mixture and mixed by vortexing for 30 s followed by 5 min of stirring before removing the hexane layer to a new container. This step was repeated three times and the mixture was centrifuged at 3500 rpm for 10 min, and the top layer was added to the same container. As a negative control, hexane by itself (without IL pretreatment) using the same process as above was only capable of extracting 6.5 ± 0.7% (*w*/*w*) oils.

### 2.5. IL Recycling

[C_2_mim][EtSO_4_] as well as [P_4444_][Prop] were tested in triplicate for the extraction performance after recycling using freeze-dried T18 as follows: 0.10 g of T18 was added at a ratio of 1:4 and 1:10 mass ratio of dry equivalent T18 to [C_2_mim][EtSO_4_] and [P_4444_][Prop], respectively. They were incubated with agitation for 1 h at ambient temperature. Lipids were extracted with hexane as previously described. After three extractions, IL was recovered by adding 5 mL of MeOH to precipitate dissolved solids followed by filtration using a fine porosity Buchner funnel. The recovered ionic liquid/methanol phase was then transferred to a round-bottom flask and the methanol was removed using evaporation with a rotary vacuum evaporator (Büchi, Flavil, Switzerland) at 150 rpm, 200 mbar, and 70 °C for 20 min, or until there was no further solvent removal. This experiment was repeated five times to determine if performance was greatly affected with repeated use. This method was normalized to the extraction yield obtained in the first use with previously unused IL.

### 2.6. Lipid Composition

FAME was prepared by dissolving 100 mg of extracted oil in 10 mL of hexane followed by the addition of 100 μL of 2 M methanolic KOH. Samples were vortexed for 30 s, followed by centrifugation, and 500 μL of the clear supernatant was spiked with the internal standard methyl undecanoate (Sigma) and separated on a Agilent 7890 Series GC equipped with a flame ionization detector (GC-FID) (Agilent Technologies Canada Inc., Mississauga, ON, Canada). The FAME mixture was separated using an Agilent DBWAX capillary column (30 m, 0.25 mm, 0.25 μm) with helium as the carrier gas at a linear velocity of 30 cm/s. Samples were injected in split mode (50:1). The FID detector was operated at 280 °C, and FAMEs were eluted using the following program: 50 °C for 1 min, 10 °C min^−1^ to 200 °C, 3 °C min^−1^ to 220 °C, 220 °C for 10 min. Individual FAMEs were quantified and identified using analytical standards (Sigma) and the internal standard C11:0. Unidentified FAMEs were estimated using an averaged RF factor.

(1) $$Oil\;Yield\;\left(\%\right)=\frac{Extracted\;Oil\;\left(mg\right)}{mass\;of\;T18\;\left(mg\right)\times maximum\;Oil\;\left(wt\%\right)}\times 100\%$$

## 3. Results and Discussion

**Figure 2:**
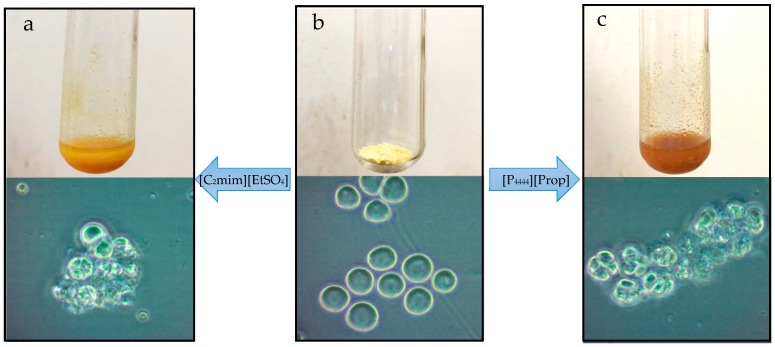
Lysis of freeze-dried T18 after the pretreatment with ionic liquids as visualized under a standard bright-field microscope; (**a**) after [C_2_mim][EtSO_4_] pretreatment, (**b**) original material, (**c**) after [P4444][Prop] pretreatment.

To confirm that [C_2_mim][EtSO_4_] and [P_4444_][Prop] were able to disrupt the cell walls of T18, images of T18 with or without IL pretreatment were captured using an optical microscope, as shown in Figure 2. Freeze-dried T18 is a light-yellow powder due to a lack of chloroplast (Figure 2b). Its cellular morphology demonstrated that these cells were intact before the pretreatment of ILs (Figure 2b). After pretreatment with [C_2_mim][EtSO_4_] (Figure 2a) and [P_4444_][Prop] (Figure 2b) for 1 h, the mixtures were observed for cell disruption. The photos of T18 treated with [C_2_mim][EtSO_4_] (Figure 2a) show that these cells were broken. Similar results were seen for treatment with [P_4444_][Prop] (Figure 2c). 

### 3.1. IL Extraction of Oils from Dried T18.

First, to determine the theoretical maximum oil content of dried T18 biomass, hexane/2-propanol (H2P) extractions were performed [40]. Dried T18 was found to contain 60.8 ± 3.9% (*w*/*w*) oils.

**Figure 3:**
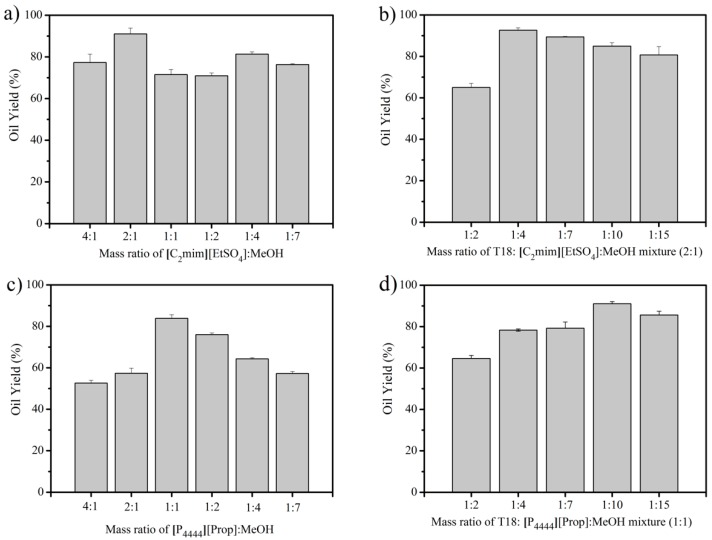
Extraction of dried T18 using [C_2_mim][EtSO_4_]: (**a**) the effect of methanol to mass ratio on the extraction of oils from dewatered T18, (**b**) the effect of increasing mass ratios of T18 to the IL/MeOH mixture (2:1) on the extraction of dried T18 using [P_4444_][Prop], (**c**) the effect of methanol to mass ratio on the extraction of oils from dewatered T18, and (**d**) the effect of increasing mass ratios of T18 to the IL/MeOH mixture (1:1). The data represent the mean of triplicates ± standard deviation.

Previous results indicated that in some cases, a co-solvent was required to increase lipid recoveries from *Chlorella vulgaris* [20]. To see if this was the case for dried T18 biomass, [C_2_mim][EtSO_4_] was mixed with the co-solvent, methanol (MeOH) at different ratios from 4:1 to 1:7 (Figure 3a), and oils were separating by briefly extracting with hexane for 5 min three times. For food applications, the use of methanol might not be suitable and preliminary results indicate that the food-grade ethanol can be used instead without affecting the performance. To compare, untreated T18 was subjected to the same extraction and only 6.5 ± 0.7% (*w*/*w*) oil content was extracted in this manner. With IL treatment, the oil yield increased until around 91.0 ± 2.8% of the theoretical maximum when the IL/MeOH ratio was 1:4. However, ratios of MeOH greater than 2:1 had a negative effect on yield, decreasing to as low as 81.3 ± 1.1%. The ratio of dried T18 biomass to the mixture of 2:1 IL/MeOH was further studied to minimize the required amount of solvent per gram of dried biomass (Figure 3b). It was found that a mass ratio of 1:4 had the best oil extraction efficiency with 92.6 ± 1.2%. Reducing the solvent to a 1:2 ratio was not enough to fully immerse the T18 biomass in the tube. However, increasing the solvent (1:7) may have caused less direct shear stress when stirring the T18 solvent mixture in the tubes. 

[P_4444_][Prop] was similarly assessed for comparison. The effect of the mass ratio of IL to MeOH (Figure 3c), and subsequently the mass ratio of T18 to the solvent system (Figure 3d), were studied. The ratios of IL to MeOH were varied from 4:1 to 1:7 using a mass loading ratio of 1:4 for T18 to solvent (Figure 3c). Oil yields increased to around 83.9 ± 1.7% when using a 1:1 ratio and then decreased in either direction. The mixture of 1:1 IL/MeOH was further characterized to optimize the ratio of T18 biomass to solvent (Figure 3d). It was found that more [P_4444_][Prop] (1:10) than [C_2_mim][EtSO_4_] (1:4) was required to achieve similar oil extraction yields of 91.0 ± 1.1%.

### 3.2. IL Extraction of Oils from Wet T18 

**Figure 4:**
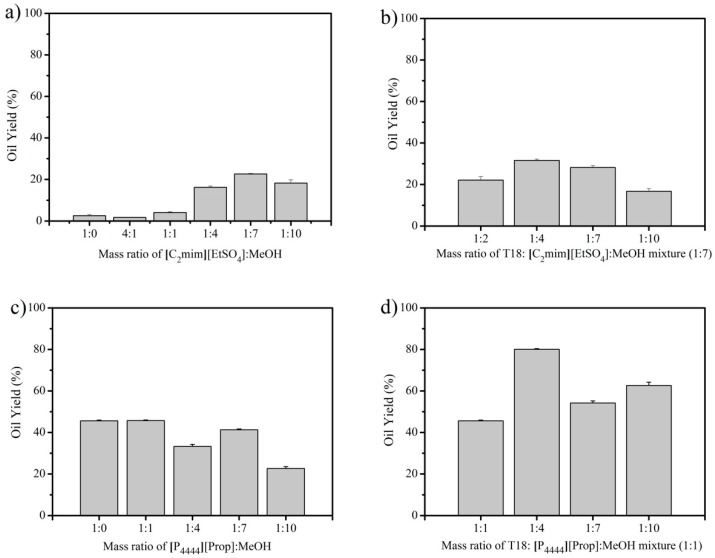
Extraction of wet T18 using [C_2_mim][EtSO_4_]: (**a**) the effect of methanol to mass ratio on the extraction of oils from wet T18 slurry, (**b**) the effect of increasing mass ratios of T18 to the IL/MeOH mixture (1:7) on the extraction of wet T18 using [P_4444_][Prop], (**c**) the effect of methanol to mass ratio on the extraction of oils from wet T18 slurry, and (**d**) the effect of increasing mass ratios of T18 slurry to the IL. The data represent the mean of triplicates ± standard deviation.

As in the previous section, the initial oil content of wet biomass was determined via H2P extraction and the batch contained 71.1 ± 2.8% (*w*/*w*) of oil. When the [C_2_mim][EtSO_4_] process was applied to wet T18, the IL extraction worked poorly, extracting only a small fraction of the available lipids (Figure 4a). Therefore, the IL/MeOH ratio was reassessed. The amount of extracted oil was the highest at the ratio of 1:7, but the maximum oil yield was still only 22.6 ± 0.3%. Since the optimal conditions were not consistent with those determined for dry biomass, the next step was to re-evaluate the loading ratio of T18 slurry to the IL/MeOH mixture (Figure 4b). Here the best ratio was again 1:4, which could recover 31.6 ± 0.7% of the oil. Thus, this ionic liquid was not a suitable candidate for wet extraction. In contrast, the optimal ratio of IL/MeOH did not change with wet T18 biomass processed with [P_4444_][prop] (Figure 4c); however, the solid loading ratio of biomass to solvent decreased from 1:10 to 1:4 (Figure 4d). 

Under these conditions, oil from T18 could be readily extracted from wet slurry with yields up to 80.1 ± 0.4%. The results show that the IL is a promising candidate for wet extraction from T18. 

### 3.3. Composition of Extracted Lipids

**Figure 5:**
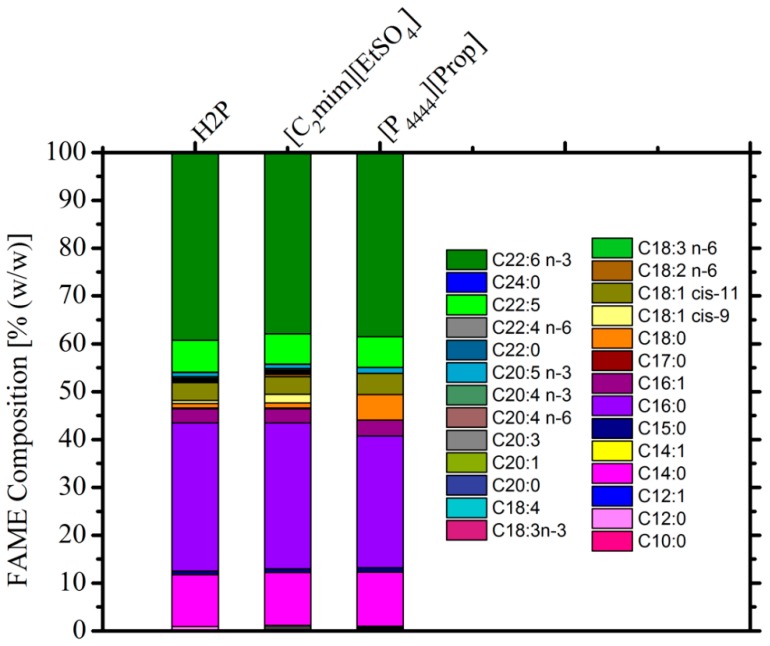
Comparison of the relative FAME composition of the oil extracted using H2P, [C_2_mim][EtSO_4_], and [P_4444_][prop]. Fatty acids are represented by the number of carbons in their chain followed by the number of unsaturated C–C bonds and the carbon number of the first unsaturated bond (e.g., C20:4n-6 represents a C20 chain with four unsaturated bonds, the first one occurring at carbon 6).

To analyze the fatty acid composition of extracts, the extracted oils were transesterified to FAME and quantified using a GC-FID (Figure 5). The FAME compositions using [C_2_mim][EtSO_4_] were no different from those extracted using the H2P method. However, the composition of the lipids recovered using [P_4444_][prop] were slightly different. The lipid extracted in this way had a greater proportion of saturated fatty acids such as C18:0. The largest constituent in all cases was C22:6n-3 (DHA), confirming that T18 was a good source of DHA. The results further show that the ILs applied in this study did not selectively extract DHAs, but extract the overall available lipids without oxidizing the unsaturated bonds, hence not compromising the quality of the SCO. 

### 3.4. Ionic Liquid Recycling

**Figure 6:**
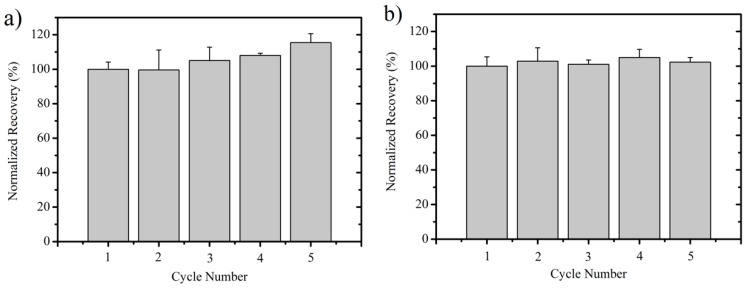
Lipid recovery with recycled [C_2_mim][EtSO_4_] (**a**), and [P_4444_][prop] (**b**), from dried T18. The data represent the mean of triplicates ± standard deviation.

To reduce the cost of the IL and improve the economic feasibility of a potential process, reusability of the two ILs was assessed. Ionic liquids were recovered from dried T18 after oil extraction by antisolvent precipitation of the residual biomass using MeOH. After residual solids were separated using filtration, residual MeOH was evaporated using a vacuum evaporator. The pretreatment performance of the recovered IL toward dried T18 oil extraction was observed for potential performance fluctuations. Figure 6 shows that the amount of oil recovered by the recycled ILs, [C_2_mim][EtSO_4_], and [P_4444_][prop] over five cycles did not change. The average oil recoveries by [C_2_mim][EtSO_4_] and [P_4444_][prop] were 98.5 ± 3.7% and 97.2 ± 2.5%, respectively. It is likely that impurities carried over between cycles did not negatively affect the oil recovery, which illustrates that both ILs possess excellent recyclability. 

## 4. Conclusions

The goal of this work was to assess the ability of ILs to aid in the extraction of thraustochytrids in a sustainable and recyclable process for DHA production. Two ILs were characterized under a series of conditions followed by lipid composition analysis. The results demonstrate that while [C_2_mim][EtSO_4_] and[P_4444_][prop] aid with the extraction of over 90% (*w*/*w*) of the available lipids from dried T18 biomass, wet T18 slurry was much more difficult to process. However, [P_4444_][prop] achieved a lipid yield of over 80% (*w*/*w*), making it a promising candidate for further studies. One significant concern when using ILs for possible food processing applications will be the toxicity of the processing agents. The co-solvent methanol can easily be replaced by food-grade ethanol. The ILs used in this work are strongly hydrophilic, mitigating the concerns to some extent, but the toxicity of these ILs will need to be evaluated moving forward. The deliberate choice of hydrophilic ILs is expected to mitigate safety concerns as the ILs and the lipid fraction are non-miscible. The lipid composition by FAME analysis showed that the major component of T18 oil was DHA, a common component for this strain. Moreover, both ILs were readily recycled with no decrease in performance over four cycles. In contrast to conventional extraction and cell disruption methods with require dried biomass, this work demonstrates a simple process that is compatible with wet T18 biomass. 

## Figures and Tables

**Table 1:** Ionic liquid-based extraction of lipids and PAF from algae.

Algal Species	Operating Conditions	Extraction Solvent/Method	Yield	Reference
*Chlorella* sp.	ILs: cells, 10:1 (*w*/*w*). Incubated for 24 h at room temperature with constant low speed stirring.		Oil yield (mg/g algae)	[28]
		Bligh and Dyer	38.13	
		Butyrolactam formate	48.0 ± 0.4	
		Butyrolactam acetate	39.1 ± 5.0	
		Butyrolactam hexanoate	46.8 ± 5.8	
		Caprolactam formate	36.3 ± 6.8	
		Caprolactam acetate	38.3 ± 4.5	
		Caprolactam hexanoate	42.9 ± 2.0 PAF 15.4 ± 2.6	
		Propylammonium formate	15.4 ± 2.6	
		Propylammonium acetate	12.8 ± 4.9	
		3-Hydroxypropylammonium formate	8.1 ± 2.1	
		3-Hydroxypropylammonium acetate	10.1 ± 1.2	
*Chlorococcum* sp.	ILs: cells, 10:1 (*w*/*w*). Incubated for 24 h at room temperature with constant low speed stirring.	Bligh and Dyer	11.55 mg/g	[28]
		Butyrolactam formate	36.4 ± 1.4	
		Butyrolactam acetate	44.4 ± 1.8	
		Butyrolactam hexanoate	51.1 ± 1.9	
		Caprolactam formate	45.7 ± 2.5	
		Caprolactam acetate	49.1 ± 2.3	
		Caprolactam hexanoate	46.7 ± 2.0	
		Propylammonium formate	18.9 ± 6.3	
		Propylammonium acetate	16.7 ± 3.2	
		3-Hydroxypropylammonium formate	13.4 ± 4.7	
		3-Hydroxypropylammonium acetate	5.9 ± 2.7	
*Aurantiochytrium* sp.	FeCl_3_ H_2_O:[Emim]OAc, 5:1 (*w*/*w*). 90 °C for 60 min (5% *Aurantiochytrium* sp. loading, *w*/*w*)		DHA content (mg/g lipid)	[29]
		Bligh and Dyer	235.2	
		Hexane	259.4	
		Hexane:Methanol = 7:3	275.7	
		1-ethyl-3-methyl imidazolium acetate	301.3	
*Chlorella vulgaris*	*C. vulgaris* (500 mg) was mixed with a mixture of 2.5 mL IL and 2.5 mL methanol under magnetic stirring at 65 °C for 18 h.		Lipid contents (%)	[27]
		Bligh and Dyer	11.1	
		[Bmim][CF_3_SO_3_]	19.0	
		[Bmim][MeSO_4_]	17.4	
